# Effects of 8 weeks pre-workout dietary supplement ingestion with and without synephrine on blood chemistry panel

**DOI:** 10.1186/1550-2783-12-S1-P4

**Published:** 2015-09-21

**Authors:** YP Jung, R Dalton, C Rasmussen, P Murano, CP Earnest, RB Kreider

**Affiliations:** 1 Exercise & Sport Nutrition Lab, Texas A&M University, College Station, TX, USA; 2 Institute for Obesity Research & Program Evaluation, Texas A&M University, College Station, TX, USA; 3 Nutrabolt International Inc., Bryan, TX, USA

## Background

A number of nutritional strategies have been developed to optimize nutrient delivery prior to exercise. As a result, a number of pre-workout supplements have been developed to increase energy availability, promote vasodilation, and/or positively affect exercise capacity. The purpose of this study was to examine the effects of 8 weeks pre-workout dietary supplement ingestion with and without synephrine on blood chemistry panel.

## Methods

In a double-blind, randomized and placebo-controlled manner; 80 apparently healthy and resistance-trained men (21.76 ± 3.59 yr, 15.29 ± 6.19% fat, 25.60 ± 4.03kg/m^2^) ingested in a randomized and counterbalanced manner a dextrose flavored placebo (P); a pre-workout supplement (PWS) containing 3.0 g beta alanine, 2 g creatine nitrate, 2g arginine AKG, 300mg N-acetyl tyrosine, 270mg caffeine, 15mg Mucuna pruriens; or, the PWS with 20mg synephrine (PWS+S), and then had blood donation at week 0, week 4, and week 8. The participants had resistance training 4 times per week during 8 weeks supplementation. Data were analyzed by repeated measure ANOVA and presented as mean (95% CI) delta change from baseline.

## Results

Repeated MANOVA revealed no significant differences among groups in blood urea nitrogen (BUN) (p = 0.62) and creatinine (CRE) (p = 0.27), and the ratio of BUN/CRE (BCr) (p = 0.20). An overall Wilks' Lambda analysis showed significant time effects (p < 0.01) in mean changes in BUN (unit conversion to mg/dl by mmol/l × 2.8011) (2.79mg/dl; 1.58, 4.00) at week 8, CRE (unit conversion to mg/dl by µmol/l × 0.0113) (-0.35mg/dl; -0.49, -0.21) at week 4 and (-0.16mg/dl; -0.28, -0.05), and BCr: (8.17; 4.01, 12.33) at week 4 and (7.02; 3.02, 11.02) at week 8. Greenhouse-Geisser univariate analysis revealed no time × group interaction of BUN (p = 0.54), CRE (p = 0.78), and BCr (p = 0.62). In liver enzymes, there were no significant differences among groups in alkaline phosphatase (ALP) (p = 0.24), alanine amino transferase (ALT) (p = 0.74), and aspartate amino transferase (AST) (p = 0.47). Delta analysis revealed significant difference in ALP: (-11.23 U/L; -13.93, -8.5) at week 4 and (-5.44 U/L; -8.48, -2.4) at week 8. LSD Post hoc analysis revealed no significant mean changes in liver enzymes; however, there was a significant difference (p = 0.04) of ALP between PWS+S (-3.44 U/L; -6.52, -0.36) and PWS (-7.86 U/L; -10.88, -4.84) compared with P (-5.36 U/L; -8.388, -2.34). However, the range of both groups PWS+S: (68.14 ± 17.39 U/L) at week 4 and (74.44 ± 19.64 U/L) at week 8 and PWS: (87.20 ± 24.72 U/L) at week 4 and (78.49 ± 24.96 U/L) at week 8 were within safe clinical range (30-92 U/L). There were no significant time (p = 0.23) and time × group interaction (p = 0.78) of creatine kinase (CK) and lactate dehydrogenase (LDH), no significant time × group interaction (p = 0.78) of total cholesterol, LDL-C, HDL-C and triglyceride, and a significant time effect (p < 0.01) but no time × group effect (p = 0.083) of glucose levels.

## Conclusion

Ingesting a dietary PWS or PWS+S for 8 weeks had no adverse effect on kidney function, liver enzymes, blood lipid levels, muscle enzymes, and blood sugar levels. These findings are in agreement with other studies testing similar ingredients.

